# Learning the heterogeneous representation of brain's structure from serial SEM images using a masked autoencoder

**DOI:** 10.3389/fninf.2023.1118419

**Published:** 2023-06-08

**Authors:** Ao Cheng, Jiahao Shi, Lirong Wang, Ruobing Zhang

**Affiliations:** ^1^School of Electronic and Information Engineering, Soochow University, Suzhou, China; ^2^Jiangsu Key Laboratory of Medical Optics, Suzhou Institute of Biomedical Engineering and Technology, Chinese Academy of Sciences, Suzhou, China; ^3^Hefei Comprehensive National Science Center, Institute of Artificial Intelligence, Hefei, China

**Keywords:** neural segmentation, SEM image, masked autoencoder, image segmentation, self-supervised learning

## Abstract

**Introduction:**

The exorbitant cost of accurately annotating the large-scale serial scanning electron microscope (SEM) images as the ground truth for training has always been a great challenge for brain map reconstruction by deep learning methods in neural connectome studies. The representation ability of the model is strongly correlated with the number of such high-quality labels. Recently, the masked autoencoder (MAE) has been shown to effectively pre-train Vision Transformers (ViT) to improve their representational capabilities.

**Methods:**

In this paper, we investigated a self-pre-training paradigm for serial SEM images with MAE to implement downstream segmentation tasks. We randomly masked voxels in three-dimensional brain image patches and trained an autoencoder to reconstruct the neuronal structures.

**Results and discussion:**

We tested different pre-training and fine-tuning configurations on three different serial SEM datasets of mouse brains, including two public ones, SNEMI3D and MitoEM-R, and one acquired in our lab. A series of masking ratios were examined and the optimal ratio for pre-training efficiency was spotted for 3D segmentation. The MAE pre-training strategy significantly outperformed the supervised learning from scratch. Our work shows that the general framework of can be a unified approach for effective learning of the representation of heterogeneous neural structural features in serial SEM images to greatly facilitate brain connectome reconstruction.

## 1. Introduction

Three-dimensional segmentation of neural structures in serial scanning electron microscope (SEM) images is a fundamental task in brain connectomics studies (Kasthuri et al., 2015; Eberle et al., 2018). Although supervised deep learning methods, such as U-Net (Ronneberger et al., 2015), have become the preferred approach for image reconstruction, they rely on annotated data, which can be costly and time-consuming for large-scale image tasks.

As a feasible alternative, self-supervised learning acquires supervised information from the data itself and has recently been shown to successfully address the need for data and be able to learn dense representations of the input (Hung et al., 2018; Lin et al., 2020; He et al., 2021; Mittal et al., 2021). For the pretext tasks, masked image modeling is such a pre-training learning task to enhance the representation capability: mask part of the input information and try to predict the masked information. This paradigm has been very successful in NLP, as self-supervised learning algorithms based on masked language modeling tasks have revolutionized the discipline. Methods such as BERT (Devlin et al., 2019) and GPT (Radford et al., 2018, 2019) have demonstrated that they can learn on unlabeled text data and are suitable for a variety of applications. With the introduction of Vision Transformers (ViT) (Vaswani et al., 2017), Masked autoencoder (MAE) (He et al., 2021) is also used to enhance the representation ability of self-attention mechanism models (He et al., 2021; Wei et al., 2022; Xie et al., 2022). Following this philosophy, state-of-the-art methods based on MAE have demonstrated their effectiveness in developing vision models.

Other common self-supervised methods on downstream tasks aim to exploit existing labels for unlabeled domains. One approach is to use discriminator constraints on the spatial distribution of predictions on unlabeled images to improve model accuracy (Hung et al., 2018). Another approach uses unpaired image-to-image translation models, such as CycleGAN (You et al., 2020) on MRI images, CySGAN (Lauenburg et al., 2022) on SEM images, to domain-shift the dataset. However, regardless of the downstream task, the segmentation relies on an optimized translation model, these methods can increase the complexity of the pipeline and require additional modules for domain adaptation.

To address these challenges, we propose to use masked autoencoders (MAE) (He et al., 2021) as a pre-training strategy that is utilized for downstream 3D SEM image tasks. MAE has not yet been thoroughly investigated for 3D electron microscope images, and its feasibility in this domain is still unknown. Therefore, our objective is to explore the applicability of MAE as a unified pre-training paradigm for various 3D electron microscope image tasks and evaluate its effectiveness compared to training from scratch. Our experiments will also include an evaluation of the proposed method on publicly available datasets.

## 2. Related work

Self-supervised learning approaches focus on learning representations from unlabeled data to achieve high precision, high accuracy, and rich representations. Transfer learning from natural images is used for medical image processing regardless of differences in image scale, and task-related features. Wen et al. (2021) used medical images datasets to initialize the network, and subsequently fine-tuned the network for various medical datasets. Raghu et al. (2019a,b) showed that transfer learning from imageNet can accelerate the convergence of medical images, which is particularly useful when medical image training data is limited. In electron microscope images, transfer learning using domain-specific data can also help address domain differences and reduce labeling costs (Januszewski and Jain, 2019; Lauenburg et al., 2022). Januszewski and Jain (2019) migrated the pre-trained segmentation model to the target data without labels so that the more accurate pseudo labels of the new dataset can be obtained directly. Lauenburg et al. (2022) proposed additional self-supervised and segmentation-based adversarial objectives in addition to the two steps of domain translation and image segmentation. Although this strategy effectively improves the representation ability of the model, it requires a part of the label as a constraint, and this data is expensive and time-consuming to collect. Besides, these domain-based self-supervised learning are difficult to combine with each other. Recent improvements in self-supervised learning offer a feasible alternative, allowing specific representations to be learned to use unlabeled data, which is massive and often more accessible.

The masked autoencoder is a self-supervised learning method that learns representations from the image itself. DAE (Vincent et al., 2008, 2010) is a pioneering work in this field that presents masking as a type of noise. It develops with the MLM task in NLP, the most representative is BERT (Devlin et al., 2019). In the field of CV, such methods continue to develop and have proven effective (Pathak et al., 2016; Dosovitskiy et al., 2021; He et al., 2021; Wei et al., 2022; Xie et al., 2022). Recent methods are based on the transformer (Vaswani et al., 2017) structure, which is a self-attention-based model capable of solving image and language tasks.

## 3. Proposed method

**Figure 1:**
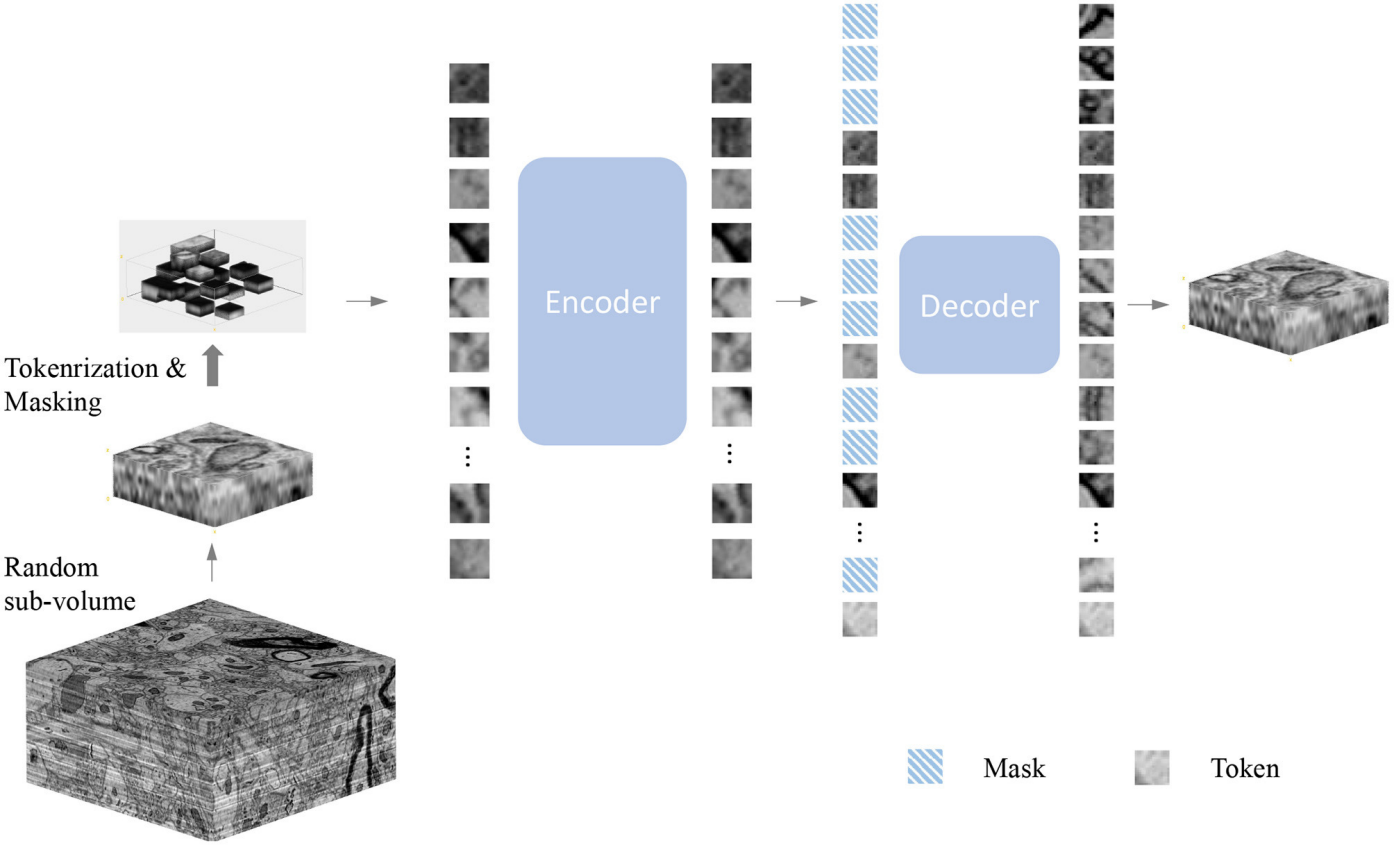
Illustration of the masked autoencoder. Firstly, we randomly sample a sub-volume from the volume dataset as training data. Next, we randomly mask voxels with a specific masking ratio after the patch embedding operation. Afterward, the encoder processes all the unmasked voxels. A smaller decoder operates the full set of voxels, which includes the masked voxels as well, outputs the final reconstruction result. Note that the value of masked voxels here is given the value 0 because our goal is to predict those masked voxels by encoded voxels.

As shown in Figure 1, our method is an extension of MAE (He et al., 2021) to 3D electron microscopy image data. Our objective is to develop methods that are applicable to electron microscopy images under a general and unified framework. Masked Image Modeling typically masks parts of the input image or encoded image tokens and promotes the model to reconstruct the masked regions. Many existing Masked Image Modeling methods employ an encoder-decoder design followed by a prediction head, such as BEiT (Bao et al., 2021) and MAE (He et al., 2021). The encoder helps to pattern the latent feature representation, while the decoder helps to process the latent features to the original image. Moreover, designing the decoder components in a lightweight size minimizes training time. In our experience, lightweight decoders not only reduce computational complexity, but also maximize the ability of the encoders to learn more general representations. In this work, we thoroughly investigate the effectiveness of different MAE models on 3D SEM image data. The following components provide more details:

### 3.1. Patch embedding

Following the original ViT (Dosovitskiy et al., 2021), given a patch, we divide it into a regular grid of non-overlapping blocks in space. These patches are flattened and embedded by linear projection (Dosovitskiy et al., 2021). The positional embedding (Vaswani et al., 2017) is added to the embedded token. The token and position embedding process is the only voxel-wise aware process. Unlike the 2D MAE (He et al., 2021) design, due to the different spatial resolutions during imaging, we do not use down-sampling in the z-direction, which ensures the 3D resolution of the voxel is close to a cube.

### 3.2. Masking

**Figure 2:**
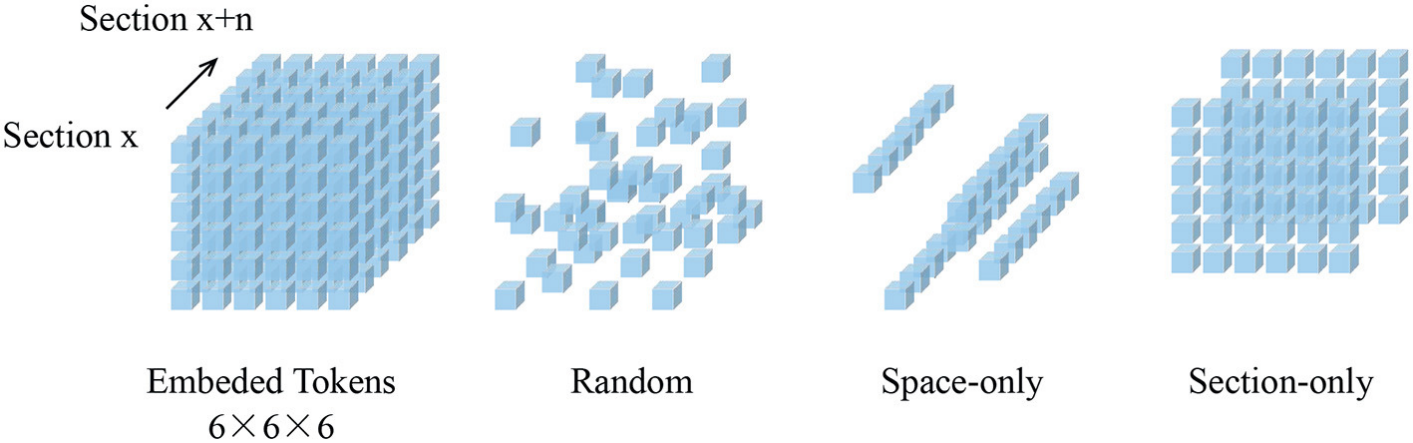
Demonstration of mask sampling strategies. In this illustration, our embedded dimension (Z × X × Y) is 6 × 6 × 6. Blue cubes represent embedded tokens. Random sampling is an agnostic spatial-wise sampling strategy. Space-only random sampling masks the tokens to all sections, and section-only random sampling masks random sections.

We randomly sample patches from the embedded patch set without replacement. This random sampling is independent of spatial structure. As shown in Figure 2, the structure-independent random sampling strategy is similar to the one-dimensional (Devlin et al., 2019) and two-dimensional (He et al., 2021; Wei et al., 2022) methods. In He et al. (2021), it is assumed that the optimal masking ratio is related to the information redundancy of the data. For unstructured random masks, BERT (Devlin et al., 2019) uses a masking ratio of 15% for languages, while MAE (He et al., 2021) uses a masking ratio of 75% for images, indicating that images are more information redundancy. Our experimental results on patch data support this hypothesis. The best masking ratio we observed for 3D MAE (He et al., 2021) on SEM images can reach 90%. This is consistent with the general assumption that the 3D SEM data are spatially coherent and more informative.

**Figure 3:**
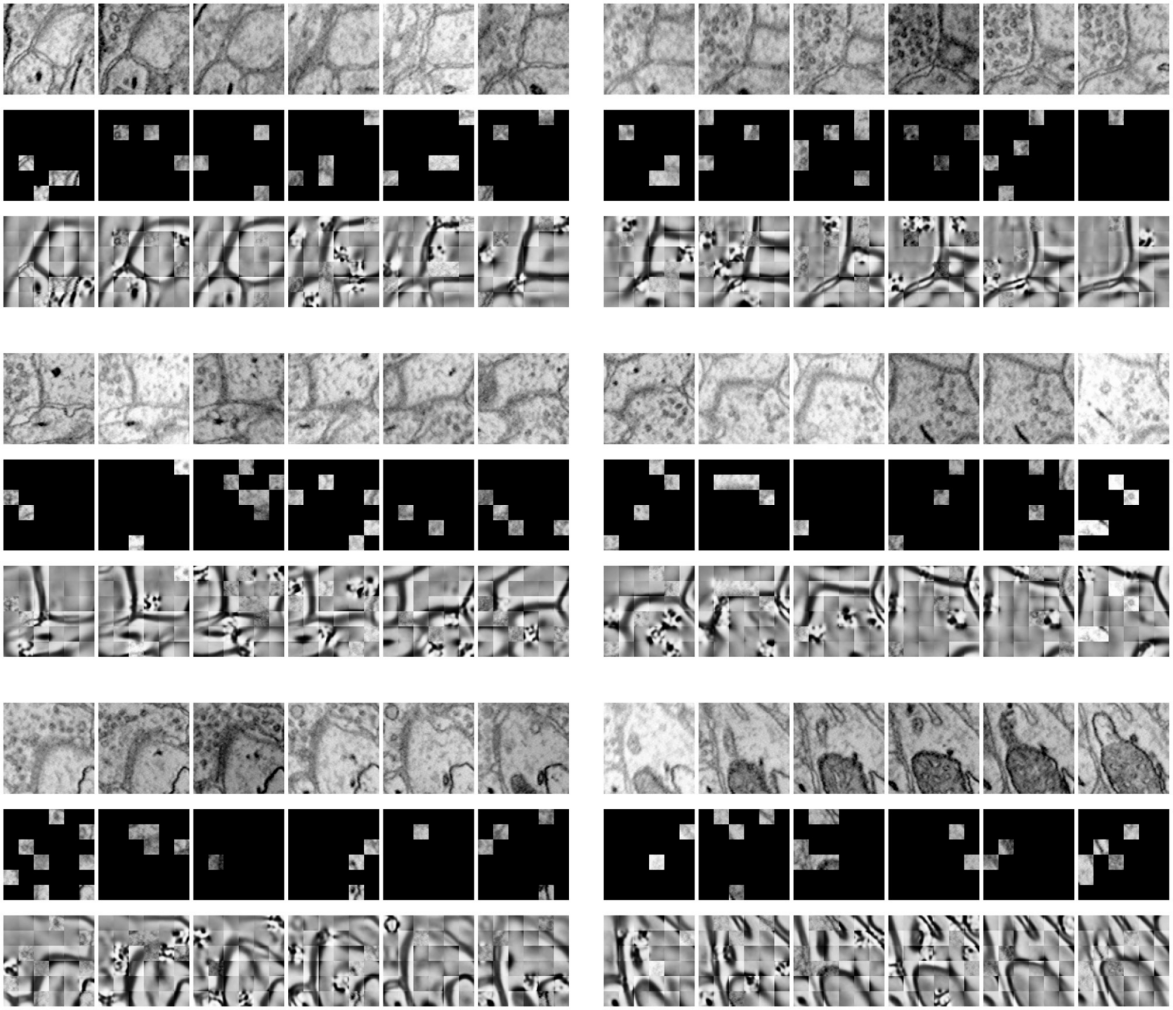
Demonstration of masked results on the SNEMI3D dataset (Lee et al., 2017). Each row represents the image volume **(top)**, masked volume **(middle)**, and the final prediction **(bottom)**. The image volume has a size of 6 × 96 × 96 and is embedded with patch size 1 × 16 × 16. After patch embedding, we obtain 216 tokens in total with the shape of 6 × 6 × 6. Since the masking ratio is up to 90%, there are only 21 visible tokens for further encoding.

Figure 3 shows the results of our MAE reconstructing the masked data, with a masking ratio of 90%. Spatial random sampling may be more efficient than structure-aware sampling strategies. Since voxels are coherent, with a very high masking ratio, space-only or slice-only sampling may retain less information and produce an overly difficult pre-training task. For example, 83.3% masking ratio with the slice-only sampling of embedded dimension 6 × 6 × 6 means that only one slice is maintained, which presents an extremely challenging task of predicting other sections. We observe that the optimal masking ratio for structure-aware sampling is generally lower. In contrast, spatial random sampling has higher efficiency on the limited number of visible patches, thus allowing the use of a higher masking ratio.

### 3.3. Autoencoding

Our encoder is a vanilla ViT (Vaswani et al., 2017), applied only to visible embedded patches, following He et al. (2021). This design greatly reduces time and memory complexity and leads to a more practical solution. A masking ratio of 90% reduces the encoder complexity to 1/10. Unlike SimMIM (Xie et al., 2022), MAE's decoder is an encoded patch set and a set of masked tokens (He et al., 2021) concatenated with another set of vanilla ViT. Decoder-specific position embeddings are added to this set (He et al., 2021). Although both are Vit structures, the size of the decoder is designed to be smaller than the encoder (He et al., 2021). Moreover, the decoder handles the complete set, but it is originally less complex than the encoder. In addition, unlike the 2D MAE, we utilize 3D sin-cos similarity as our 3D MAE's positional embedding to provide information about the spatial location.

We use the decoder to predict patches in the voxel space. We follow (He et al., 2021), predicting full spatial voxels (e.g., Z × 16 × 16) and the normalized value of each block of the original voxel. The training loss function is the mean-squared error (MSE) between the prediction and its target, averaged over unknown blocks (Devlin et al., 2019). This method relies on global self-attention to learn useful knowledge from the data, following Dosovitskiy et al. (2021).

## 4. Experiment results

### 4.1. Implementation

Our encoder and decoder are the vanilla ViT architectures (Vaswani et al., 2017). We use a patch size of 1 for the z-direction's patch embedding, which follows the features of the SEM dataset. And we implement a space patch size of 16 × 16 (Dosovitskiy et al., 2021), denoted as 1 × 16 × 16. We use the same patch size for ViT-B/L (Dosovitskiy et al., 2021) for simplicity. For a 6 × 96 × 96 input, this patch embedding size produces 6 × 6 × 6 tokens and is embedded with 3D positional embeddings for further encoding.

The 3D MAE pre-training configuration on SNEMI3D (Lee et al., 2017) is shown in **Table 6**. We use the AdamW optimizer (Kingma and Ba, 2014) with a batch size of 128 on 6 NVIDIA RTX3090 GPUs. We evaluate the pre-training quality by end-to-end fine-tuning. Furthermore, we remove the pre-trained decoder and implement UNETR (Hatamizadeh et al., 2022) as our model's architecture. In the experiments of fine-tuning, we compare the different predicting targets, affinity maps (Lee et al., 2017), and multi-task predictions (Wei et al., 2020). The loss function of predicting affinity map follows the proposed configurations from Lee et al. (2017) and Lin et al. (2021). And the loss function for multi-task predictions following the configurations from Wei et al. (2020) and Lin et al. (2021). In addition, the following post-processing step for affinity maps and multi-task predictions are using the default configuration (Lin et al., 2021).

The SNEMI3D (Lee et al., 2017) leaderboard use adapted Rand F-score (A-Rand) (Rand, 1971; Nunez-Iglesias et al., 2013) as evaluation metrics. To show the significance of different methods, we demonstrated segmentation accuracy through variation of information (VI) (Bogovic et al., 2013) and adapted Rand F-score (Rand, 1971; Nunez-Iglesias et al., 2013). VI is defined as:


(1) $$\begin{array}{l} VI\left(S,T\right)=H\left(S|T\right)+H\left(T|S\right) \end{array}$$

Where *S* and *T* represents segmentation results and its related ground truth. Then the conditional entropy *H*(*S*|*T*) measures oversegmentation errors (split error), and *H*(*T*|*S*) measures undersegmentation errors (merger error). We defined split error and merge error as Voi-S and Voi-M, respectively.

### 4.2. Ablation study

In this section, we assessed the model's pre-training performance across four aspects: sampling strategy, masking ratio, decoding depth, and decoding dimension. The ultimate fine-tuned models with different sampling methods are evaluated on the SNEMI3D dataset (Lee et al., 2017). Furthermore, we exclusively used Vit-Base for all ablation experiments and the pre-training dataset is the training dataset from SNEMI3D.

Table 1 shows the masking strategy between random, space-only, and section-only sampling. For a fair comparison of the masking strategy and masking ratio, we set the decoder depth and decoder dimension to 4 and 512, respectively. Moreover, in the experiment of the masking strategy, we decided to make the masking ratio as close as possible. We demonstrate different sampling strategies in Table 1. Random sampling strategy has the best performance with the highest 90% masking ratio, which gains 0.071 of A-Rand. For the space-only sampling, we reserved 4 × 6 = 24 tokens, leading to a masking ratio up to 89%. This strategy performs close to random sampling. The masking ratio for the section-only sampling strategy is 83%, it processes one section's voxels. As shown in Table 1, the section-only sampling has the worst performance. Since this sampling strategy needs to predict the other 5 sections, It is hard to learn a general representation with the lack of information.

**Table 1:** Demonstration of mask sampling strategy.

**Mask**	**Ratio (%)**	**Voi-S**	**Voi-M**	**A-Rand**
Random	90	0.234	0.129	0.071
Space	89	0.271	0.140	0.079
Section	83	0.313	0.156	0.103
Random	50	0.253	0.131	0.081
Random	75	0.243	0.116	0.073
Random	90	0.234	0.129	0.071
Random	95	0.249	0.131	0.077

Figure 2 shows the random mask sampling results as well. Right hand side is the demonstration of masking ratios with random mask sampling. In this table, the masking ratio is increased from 50% up to 95%, and the parameters of the decoder remain unchanged.

To have a more comprehensive look at the random sampling strategy, we analyzed the impact on the mask ratio. In this part, we only change the masking ratio and keep the decoder depth and dimension into 4 and 512. Table 1 shows the influence of the masking ratio jointly with the pre-training length. The ratio of 90% works the best. Because of the information redundancy of the data, the masking ratio of the random sampling strategy can increase to 90%. Furthermore, a higher masking ratio conducts in fewer tokens encoded by the encoder, which means the training speed is faster.

Table 2 reports the influence of the decoder depth and dimension. The best decoder depth and dimension are determined to 4 and 512 respectively. As shown in this table, the accuracy is degraded by large margins when using an overly decoding architecture. In 2D MAE (He et al., 2021), the proposed decoding depth from the ablation study is 8. In our 3D task, the optimal decoder depth is 4 which is lower than the proposed depth on 2D MAE (He et al., 2021). This part is also related to the differences in information redundancy between the 2D and 3D data.

**Table 2:** Demonstration of decoder depth and dimension with random sampling strategy.

**Depth**	**Dimension**	**Voi-S**	**Voi-M**	**A-Rand**
2	512	0.264	0.157	0.083
4	512	0.234	0.129	0.071
8	512	0.256	0.143	0.079
4	128	0.325	0.173	0.089
4	256	0.317	0.166	0.085

Left and right hand side is the demonstration of decoder depth with fixed dimension 512. Right hand side is the demonstration of decoder dimension with fixed depth 4. An overlay decoder degrades the accuracy.

### 4.3. Evaluation results

#### 4.3.1. SNEMI3D

**Figure 4:**
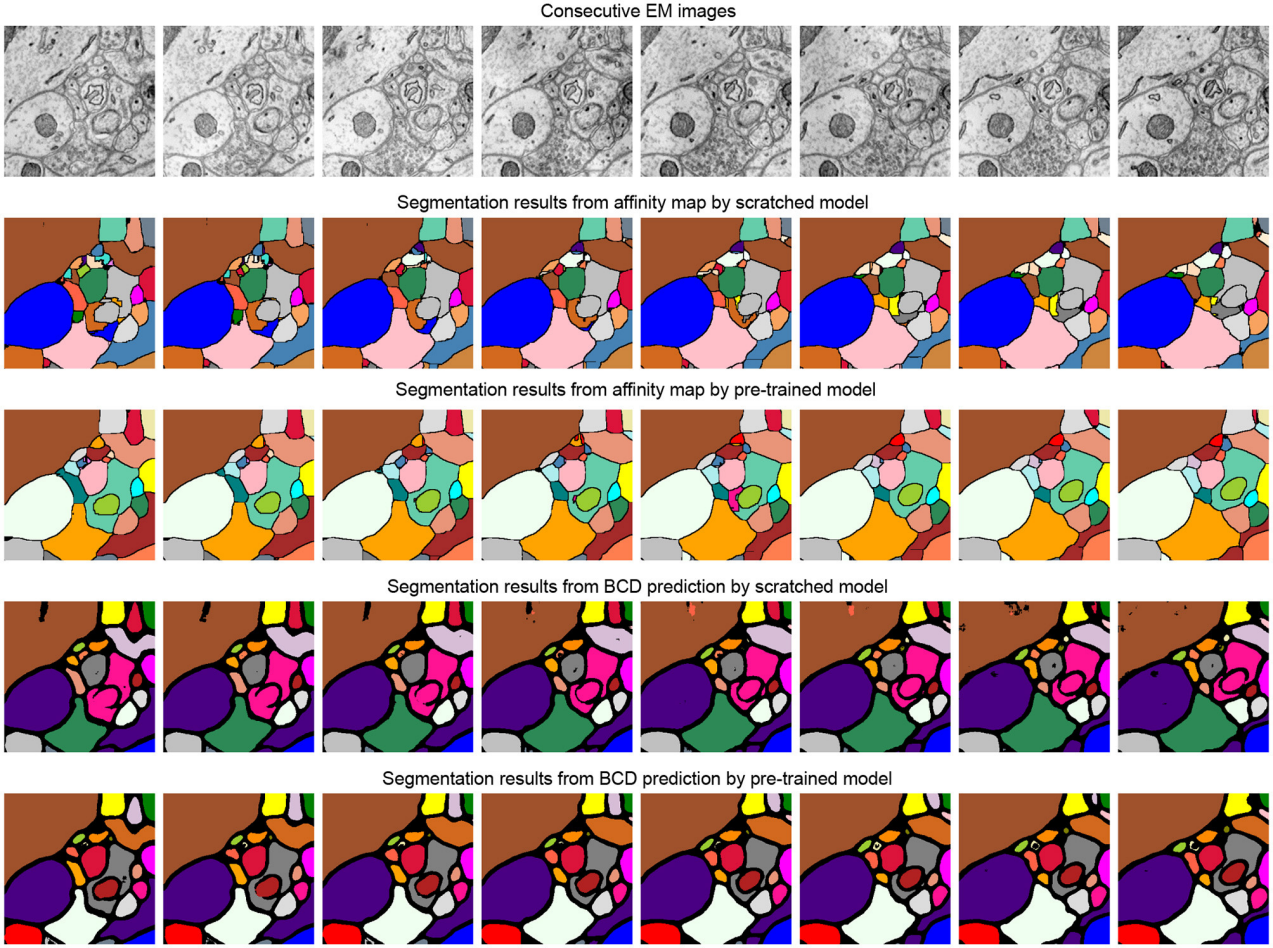
Illustration of the segmentation results on scratched and pre-trained Vit-Large. The first row is consecutive EM images from SNEMI3D (Lee et al., 2017). The second and fourth row represents the segmentation results of the model that is training from scratch, and the pre-training method results are shown in the third and fifth row. We use zwatershed as the post-processing step to generate segmentation results from the predicted affinity map. The post-processing algorithm of BCD (Binary maps, contours, distance) predictions are following the configuration from Lin et al. (2021).

Table 3 studies the differences between the pre-training strategy and training from scratch on the SNEMI3D dataset (Lee et al., 2017). Moreover, it shows the difference between predicting targets. In Table 3, for the prediction of the target, BCD prediction represents the multi-task learning method (Wei et al., 2020), which includes predicting binary maps, contours, and distance (BCD). As shown in Figure 4, we find that predicting the affinity maps presents a more accurate result on small objects, regardless of whether it is pre-trained. Moreover, evaluate metric also shows that predicting affinity maps performs better than predicting BCD. For the training method, as shown in Table 3, the pre-trained model gains comprehensive improvement on both predicting targets compared with the scratched model. The pre-trained Vit-Base and Vit-Large gain 0.071 and 0.063 of the A-Rand value, respectively. Moreover, as shown in Figure 3, regardless of the failure of high-frequency information reconstruction in MAE (He et al., 2021) pre-training, the pre-trained models outperform the scratched models.

**Table 3:** Evaluation results of SNEMI3D (Lee et al., 2017). Time and params are measured in millisecond (ms) and million (m).

**Target**	**Method**	**Backbone**	**Voi-S**	**Voi-M**	**A-Rand**	**Time**	**Params**
Affinity	Scratch	Vit-B	0.431	0.334	0.109	
	Pre-train	Vit-B	0.234	0.129	0.071	138	154
	Scratch	Vit-L	0.379	0.318	0.092
	Pre-train	Vit-L	0.211	0.106	0.063	195	455
BCD	Scratch	Vit-B	0.482	0.341	0.116
	Pre-train	Vit-B	0.331	0.215	0.084	142	154
	Scratch	Vit-L	0.415	0.301	0.095
	Pre-train	Vit-L	0.281	0.185	0.079	200	455

We also profile the parameters of the models and inferencing times in Table 3. Time and parameters are measured in millisecond (ms) and million (m). We measure the inference time of a single batch with batch size 1. Moreover, we observe that predicting the affinity map has the fastest inference time. Furthermore, the theoretical computational complexity (FLOPs) for the model with backbone of Vit-Base and Vit-Large is 154.1G and 221.1G, respectively.

#### 4.3.2. MitoEM-R

**Figure 5:**
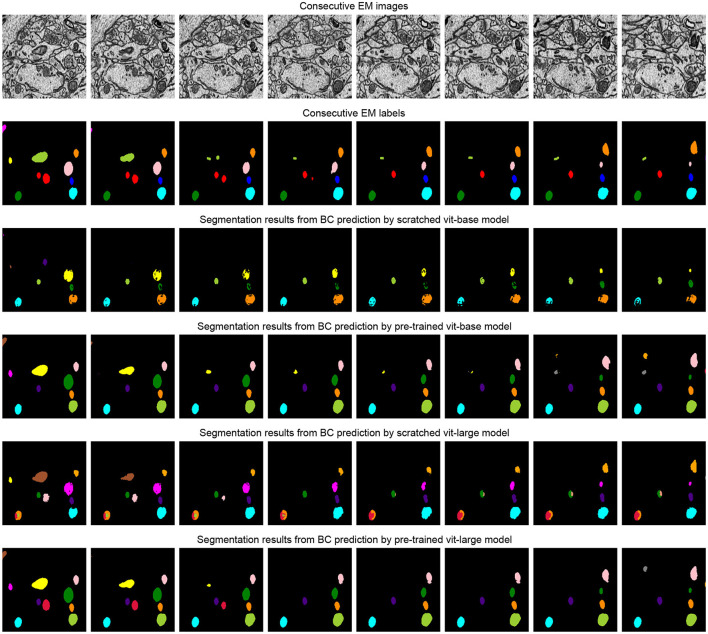
Illustration of the segmentation results on the MitoEM-R dataset (Wei et al., 2020). The first row is consecutive EM images, and the second row is its related labels. The third and fifth row represents the segmentation results of the model that is training from scratch, and the pre-training method results are shown in the fourth and sixth row. We use the watershed algorithm as the post-processing step to generate segmentation results from the predicted binary maps and contours, following the configuration from Lin et al. (2021).

The task of this dataset (Wei et al., 2020) is the instance segmentation of mitochondria. Following the same experiment settings from Wei et al. (2020), we use the binary maps and instance contours as our targets to fine-tune the models. The configurations are shown in Table 4. The post-processing steps for all the models are following the default configuration from Wei et al. (2020) and Lin et al. (2021). Moreover, we calculate the value of mAP on the validation dataset of MitoEM-R. Table 5 demonstrates the differences between the pre-training strategy and the training from scratch on the MitoEM-R dataset (Lee et al., 2017). As shown in Table 5, pre-trained Vit-Large obtains best results on both AP-75 and AP-50. Moreover, the Vit-Large with training from scratch performs worse than the pre-trained Vit-Base. It proves the MAE's (He et al., 2021) capability of representation learning on small objects such as mitochondria. Furthermore, we notice the value of AP-75 from Vit-Large has a giant improvement compared with Vit-Base. Higher AP-75 means the accurate shape and contour predictions from the model, see Figure 5, pre-trained Vit-large present the best segmentation results compared with other methods.

**Table 4:** MitoEM-R (Wei et al., 2020) fine-tuning configuration.

**Config**	**Value**
Optimizer	SGD
Weight decay	0.0001
Base learning rate	4e-3
Learning rate schedule (Loshchilov and Hutter, 2016)	Cosine decay
Warmup iteration (Goyal et al., 2017)	10,000
Dropout (Srivastava et al., 2014)	0.3
Dropout path (Huang et al., 2016)	0.1
Total iteration	300,000
Augmentation	Default by Lin et al. (2021)
Scales	[1, 0.5, 0.5]
Batch size	8
Input size	6 * 96 * 96

**Table 5:** MitoEM-R (Wei et al., 2020) evaluation results of AP-50 and AP-75.

**Method**	**Backbone**	**AP-50**	**AP-75**
Scratch	Vit-B	0.549	0.174
Pre-train	Vit-B	0.895	0.514
Scratch	Vit-L	0.797	0.431
Pre-train	Vit-L	0.923	0.679

#### 4.3.3. A white matter dataset

**Figure 6:**
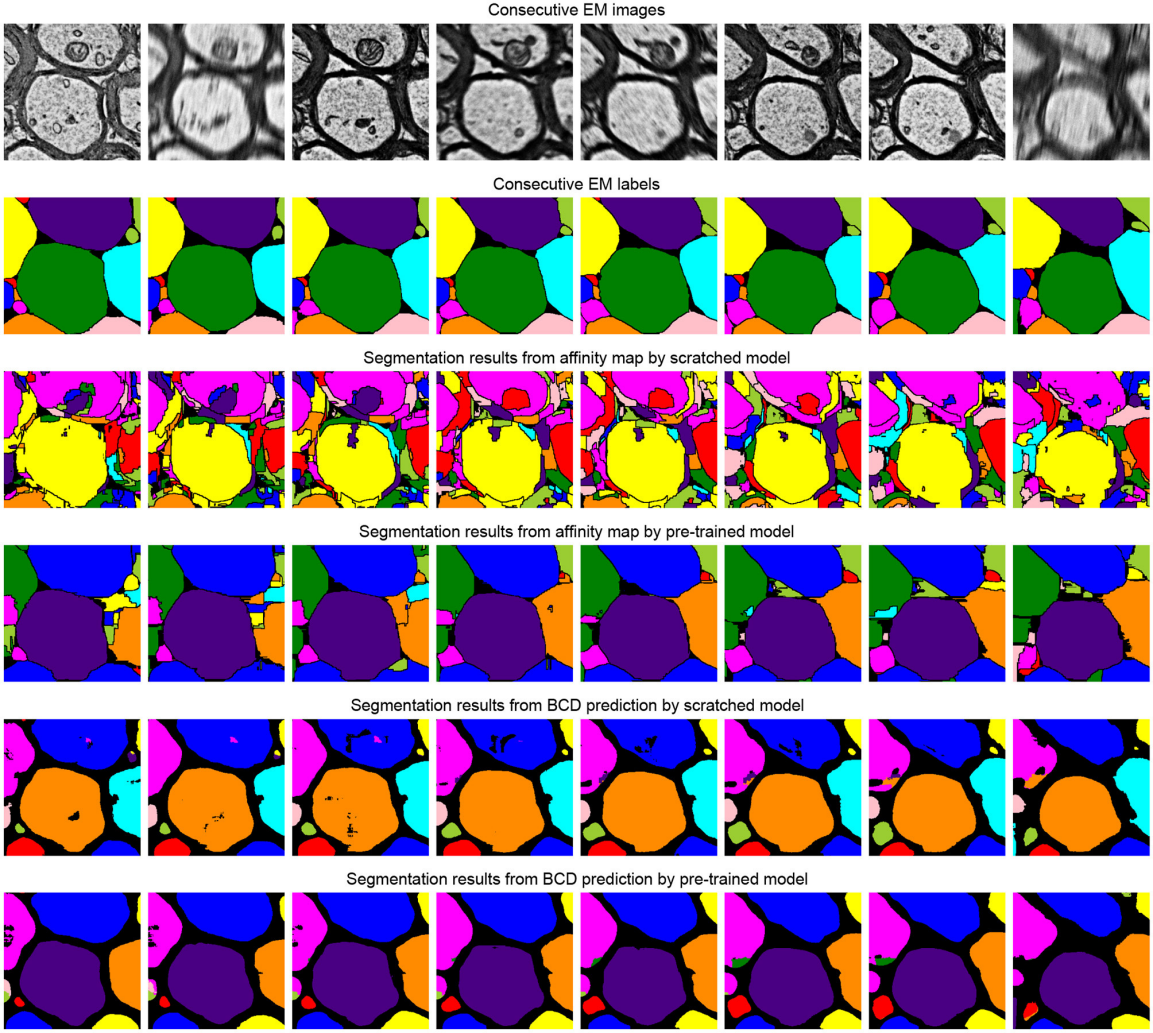
Illustration of the corpus callosum dataset segmentation results on the scratched and pre-trained Vit-Large. The first row is consecutive EM images, and the second row is its related labels. The third and fifth row represents the segmentation results of the model that is training from scratch, and the pre-training method results are shown in the fourth and sixth row. We use zwatershed as the post-processing step to generate segmentation results from the predicted affinity map. The post-processing algorithm of BCD (binary maps, contours, distances) predictions are following the configuration from Lin et al. (2021).

In this part, we demonstrate the generalization of the model that was pre-trained on SNEMI3D (Lee et al., 2017), a gray matter dataset, to fine-tune on a white matter dataset containing very different structural patterns. The fine-tuning dataset is on the region of the corpus callosum, which contains amounts of myelinated axons and some blurry sections. We manually annotated two different volumes from this dataset for further segmentation experiments. The shape of training and testing volume is 50 × 3000 × 3000 and 59 × 3000 × 3000 with the resolution of 4 nanometers per pixel, respectively. The fine-tuning process is following the same configuration for SNEMI3D (Lee et al., 2017) in Table 6. As shown in Table 7, the pre-trained model outperforms the model that trains from scratch in terms of different predicted targets. The pre-trained Vit-Large gains 0.197 of A-Rand. The visual results are shown in Figure 6. It proves the representation learning from 3D MAE (He et al., 2021) can promote model performance even when the pre-training dataset and fine-tuning dataset are enormously different. Moreover, in Figure 6, we notice the model that predicting BCD performs better than affinity prediction. Empirically, because of the additional constraining of contour prediction, it allows the model overcomes the impact of blur affections. In addition, the contours of the myelin sheath are thicker than the cell's membrane, which degrades the challenge of predicting boundaries.

**Table 6:** SNEMI3D (Lee et al., 2017) pre-training and fine-tuning configuration.

**Config (pre-training)**	**Value**
Optimizer	AdamW (Loshchilov and Hutter, 2017)
Optimizer momentum	β_1_, β_2_ = 0.9, 0.95 (Mark et al., 2020)
Weight decay	0.005
Base learning rate	1e-4
Learning rate schedule (Loshchilov and Hutter, 2016)	Cosine decay
Warmup iteration (Goyal et al., 2017)	50,000
Total iteration	400,000
Batch size	128
Input size	6 * 96 * 96
**Config (fine-tuning)**	**Value**
Optimizer	AdamW (Loshchilov and Hutter, 2017)
Optimizer momentum	β_1_, β_2_ = 0.9, 0.95 (Mark et al., 2020)
Weight decay	0.05
Base learning rate	1e-4
Learning rate schedule (Loshchilov and Hutter, 2016)	Cosine decay
Warmup iteration (Goyal et al., 2017)	5,000
Dropout (Srivastava et al., 2014)	0.3
Dropout path (Huang et al., 2016)	0.1
Total iteration	200,000
Augmentation	Default by Lin et al. (2021)
Batch size	8
Input size	6 * 96 * 96

**Table 7:** Evaluation results on the dataset of corpus callosum, which is the region of the gray matter.

**Target**	**Method**	**Backbone**	**Voi-S**	**Voi-M**	**A-Rand**
	Scratch	Vit-B	4.122	0.884	0.600
	Pre-train	Vit-B	1.407	0.378	0.214
	Scratch	Vit-L	3.426	0.678	0.316
Affinity	Pre-train	Vit-L	0.974	0.205	0.197
	Scratch	Vit-B	1.270	1.116	0.388
	Pre-train	Vit-B	1.098	1.320	0.326
	Scratch	Vit-L	0.898	0.921	0.278
BCD	Pre-train	Vit-L	0.775	0.826	0.241

## 5. Discussion

This paper proposes the paradigm of implementing MAE (He et al., 2021) to the SEM dataset, which the pre-trained Vit (Vaswani et al., 2017) can be implemented as the backbone of the UNETR (Hatamizadeh et al., 2022) for downstream tasks. We optimized the best configuration of three-dimensional MAE (He et al., 2021) pre-training for the SEM dataset. In Table 1, empirically show the efficient representation learning with the 90% random mask sampling strategy. Moreover, Table 2 proves the overlay decoder design can cause the degradation of accuracy.

**Figure 7:**
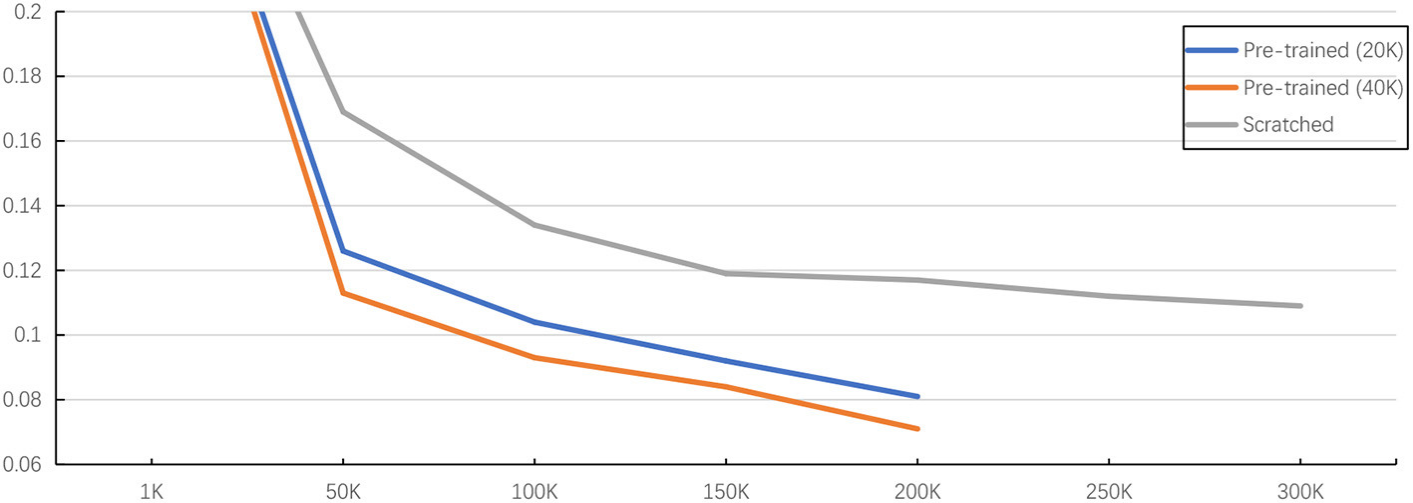
Illustration of pre-training method with fine-tuning and training from scratch. The evaluation is under the Vit-Base with predicting affinity map on SNEMI3D dataset (Lee et al., 2017). Here, x-axis is the training iterations and y-axis is value of A-Rand (Nunez-Iglesias et al., 2013). The training configuration is shown at Table 6. The pre-training method outperforms the training from scratch and more pre-training iterations prompt better representation learning even the loss is almost converged.

As shown in Table 5, the experiment results on public dataset SNEMI3D (Lee et al., 2017) illustrate the performance, parameters, and inference time of using 3D MAE (He et al., 2021) in downstream tasks. By predicting affinity map, the pre-trained Vit-Large gains 0.063 of the A-Rand, while the training from scratch method achieves 0.092 of the A-Rand. We reflect the impact of pre-training iterations, pre-training method plus fine-tuning and training from scratch in Figure 7. As shown in this figure, the pre-training with fine-tuning is much more accurate than the random initialization. Moreover, from Table 5, the experiments on MitoEM-R (Wei et al., 2020) demonstrate that pre-training on SNEMI3D (Lee et al., 2017) can also significantly enhance the performance of the tasks on MitoEM-R dataset, while pre-trained Vit-Large obtains 0.679 of AP-75. Note that the pre-training of the backbone was on SNEMI3D (Lee et al., 2017). We also discovered that the pre-trained backbone has a positive impact on the corpus callosum dataset, which is the region of the gray matter. As shown in Table 7, the pre-trained Vit-Large gains 0.197 of A-Rand compared with 0.316 of A-Rand by the training from scratch. Such enormous improvement proves the difference across datasets does not constrain the representation learning from 3D MAE (He et al., 2021). In addition, the experiments demonstrate the potential of implementing pre-trained vit (Vaswani et al., 2017) as the backbone to solve the downstream tasks.

## 6. Conclusion

We explored the paradigm of implementing MAE (He et al., 2021) to the SEM dataset. We found that representation learning for neural structure heterogeneity is possible with minimal domain knowledge. Similar to the MAE (He et al., 2021) and BERT (Devlin et al., 2019), the masking ratio is strongly related to the information redundancy of the data. Therefore, we found the time cost of the MAE pre-training paradigm for the SEM volume dataset can be tremendously reduced. We reported encouraging results of using pre-trained vit (Vaswani et al., 2017) as the backbone on two public white matter datasets, and a gray matter dataset. The pre-training method achieves strong performance and shows the capability of efficient representation learning across different structure patterns.

## Data availability statement

The datasets presented in this study can be found in online repositories. The names of the repository/repositories and accession number(s) can be found in the article/supplementary material.

## Author contributions

AC designed the research and participated in the entire research including data processing, model construction, result interpretation, and manuscript drafting. JS prepared the dataset of corpus callosum including error checks and annotations. LW and RZ designed the research and participated in the data collection and revisions of the manuscript. All authors contributed to the article and approved the submitted version.
