# Corrigendum: Learning the heterogeneous representation of brain's structure from serial SEM images using a masked autoencoder

**DOI:** 10.3389/fninf.2023.1337766

**Published:** 2023-11-27

**Authors:** Ao Cheng, Jiahao Shi, Lirong Wang, Ruobing Zhang

**Affiliations:** ^1^School of Electronic and Information Engineering, Soochow University, Suzhou, China; ^2^Jiangsu Key Laboratory of Medical Optics, Suzhou Institute of Biomedical Engineering and Technology, Chinese Academy of Sciences, Suzhou, China; ^3^Institute of Artificial Intelligence, Hefei Comprehensive National Science Center, Hefei, China

**Keywords:** neural segmentation, SEM image, masked autoencoder, image segmentation, self-supervised learning

In the published article, there was an error in the **Acknowledgements** section. We erroneously excluded contributions to this article from those who prepared the samples.

A correction has been made to the **Acknowledgements** section.

The corrected sentence appears below:

“We would like to thank Dingsan Luo and Renzheng Liu of the Jiangsu Key Laboratory of Medical Optics at the Suzhou Institute of Biomedical Engineering and Technology, Chinese Academy of Sciences, for their meticulous preparation of the mouse corpus callosum samples. We also would like to thank the Hanhua Lab from the Institute of Automation, Chinese Academy of Sciences for the corpus callosum SEM data.”

The authors apologize for this error and state that this does not change the scientific conclusions of the article in any way. The original article has been updated.

